# How to account for hallucinations in the interpretation of the antidepressant effects of psychedelics: a translational framework

**DOI:** 10.1007/s00213-022-06106-8

**Published:** 2022-03-29

**Authors:** Manon van den Berg, Igor Magaraggia, Rudy Schreiber, Todd M. Hillhouse, Joseph H. Porter

**Affiliations:** 1 Faculty of Health, Medicine and Life Sciences, Maastricht University, Maastricht, The Netherlands; 2 Faculty of Psychology and Neuroscience, Section Neuropsychology & Psychopharmacology, Maastricht University, Maastricht, The Netherlands; 3 Department of Psychology, University of Wisconsin Green Bay, Green Bay, WI USA; 4 Department of Psychology, Virginia Commonwealth University, Richmond, USA

**Keywords:** Psychedelics, Depression, Lysergic acid diethylamide (LSD), Psilocybin, *N*,*N*-dimethyltryptamine (DMT), Drug discrimination, Pattern separation (PS), Cognitive flexibility, Head twitch response (HTR)

## Abstract

**Rationale:**

Recent trials with psychedelics in major depressive disorder and treatment-resistant depression showed remarkable improvements in depressive symptoms that can last for up to several months after even a single administration. The lack of an appropriate placebo control group—as patients are often able to discriminate the subjective effects of the drug—*and* an incomplete understanding of the role of the hallucinogenic and mystical experience, hampers the interpretation of these therapeutic effects.

**Objectives:**

To control for these factors, we developed a translational framework based on establishing pharmacokinetic/pharmacodynamic (PK/PD) relationships in rodents and humans for hallucinogenic (i.e., discriminative stimulus effects in rodents and humans; head twitch responses in rodents; questionnaires in humans) and therapeutic effects. For the latter, we selected the pattern separation and attentional set-shifting tasks as measures for cognitive flexibility because of their high translational value. We predict that these PK/PD analyses will lead to a more objective evaluation of improvements in patients compared to relying only on the currently used self-reported questionnaires. We hypothesize that—if the role of the hallucinogenic experience is *not* central in the antidepressant effects of psychedelics—the ED_50_’s for the therapeutic effects will be significantly *lower* than for the hallucinogenic and mystical effects.

**Conclusion:**

Our framework will help to inform future studies that aim at the elucidation of the mechanism(s) of action of psychedelics in depression, and the role of the acute subjective and/or hallucinogenic experience in their effects.

## Introduction

Classical psychedelics, such as LSD (lysergic acid diethylamide), psilocybin, and *N*,*N*-dimethyltryptamine (DMT), are well-known for their psychoactive effects, which include perceptual changes, ego dissolution, and euphoria (Nichols 2016). From the 1950s on, it was believed that these effects could be useful in the treatment of psychiatric disorders. This idea was later confirmed by several studies in patients with trauma-related or alcohol use disorder (Dyck 2005; Osmond 1957; Sarett et al. 1966; Sessa 2016). After decades of stagnation, a renewed interest in psychedelic research has led to a series of modern randomized controlled trials (RCTs) which have provided initial evidence for the therapeutic effects of psychedelics in various depressive disorders (Carhart-Harris et al. 2016, 2021; Davis et al. 2021; Griffiths et al. 2016; Palhano-Fontes et al. 2019). In these trials, when administered under close supervision, single or double doses of psilocybin or the DMT-containing brew ayahuasca were able to induce rapid and long-lasting improvements in depressive symptoms, that in some cases even lasted up to several months after administration (Carhart-Harris et al. 2016).

**Fig. 1:**
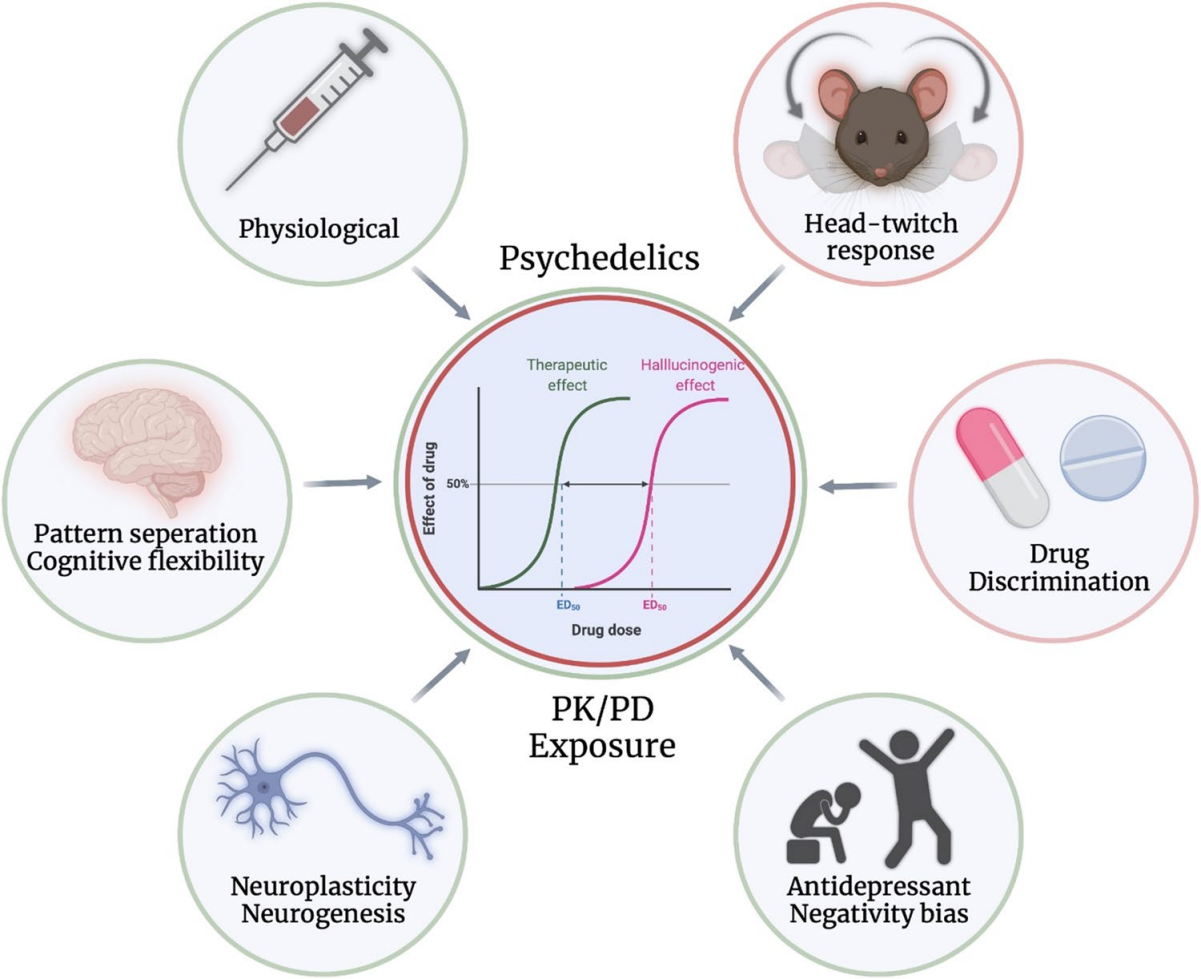
Proposed translational framework to investigate the effects of psychedelics in depression and healthy individuals. The red circles correspond with the hallucinogenic effects; the green circles with the therapeutic effects; and the double circles with both

There have been multiple clinical and preclinical investigations of the effects of psychedelics on the behavioral, cognitive, and (neuro)biological processes that are involved in the pathophysiology of depression. Yet, these investigations are limited in number, and it is still unclear how these substances work in both healthy and diseased states and there is currently no consensus on how psychedelics may exert their unique antidepressant effects. Moreover, there is a current debate regarding the role of the psychedelic and mystical experience in the presumed antidepressant effects of these drugs. In this narrative review, we focused on three classical psychedelics that that have been a primary focus of interest in clinical use—LSD, psilocybin, and ayahuasca (DMT). We searched both preclinical and clinical literature, using PubMed/Medline, clinicaltrials.gov, Google Scholar, and PsycInfo (EBSCOhost). Our aims included (1) discussing the role of the acute subjective, mystical, and hallucinogenic experience in the antidepressant effects of psychedelics; (2) summarizing what is known about the vertical (rodent vs. human) and horizontal (healthy vs. disease) translation of dose-time-dependent effects of the classical psychedelics; and (3) identifying the gaps of knowledge in translational psychedelic research, and ultimately propose a translational model that could aid and inform future studies (Fig. 1).

## Basic pharmacology

Serotonergic psychedelics include a vast list of compounds that can be divided in two main groups based on their chemical structure: tryptamines and phenethylamines (M. W. Johnson et al. 2019; Zawilska et al. 2020). Among these, LSD, psilocybin, and DMT are known as classical psychedelics (Nichols and Walter 2021). We selected LSD as our benchmark as this is the most widely studied and best characterized psychedelic. Psilocybin was selected since most recent clinical studies in the psychedelic field seem to be conducted with this compound (see sections below). Finally, we chose ayahuasca/DMT as the “psychedelic of the future,” as this drug is increasingly used for human studies. For the sake of brevity, we will simply refer to these compounds as psychedelics. As for all other serotonergic psychedelics, these substances act primarily as (partial) agonists of the serotonin_2A_ (5-HT_2A_) receptor, yet they also show significant 5-HT_1A_ receptor agonistic activity (Banks et al. 2021; Nichols 2016). Importantly, human studies with psilocybin and LSD often administered these substances in a pure synthetic form, whereas the effects of DMT have been tested mostly in the form of the Amazonian brew ayahuasca. An important distinction must be made with regards to the pharmacology of ayahuasca. Besides DMT, this brew contains the β-carboline derivative alkaloids harmine, harmaline, and tetrahydroharmine (Frecska et al. 2016). These compounds act as monoamine oxidase inhibitors (MAOIs) that block the degradation of DMT in the human body, thus increasing its bioavailability and elimination half-life (Domínguez-Clavé et al. 2016). MAOIs also block the degradation of other monoamine neurotransmitters, such as serotonin and epinephrine, complicating the study of the effects of ayahuasca and the role of DMT in its effects in humans (for more information about ayahuasca, please refer to Simão et al. (2019). Despite these differences in their pharmacodynamic profile, the shared mechanisms of 5-HT_2A_ receptor activation stands in favor of its fundamental role in the subjective and physiological properties of LSD, psilocybin, and DMT/ayahuasca.

Current research with psychedelics has mainly focused on the effects of high, hallucinogenic doses (see sections below). Recently, there is a growing interest in the use of the so-called psychedelic “microdosing,” which refers to the intermittent self-administration of sub-hallucinogenic doses of psychedelics, mostly LSD or psilocybin, for a duration that can span from 2 weeks to several months. However, the term “microdosing” should be used with caution as clear and commonly accepted criteria for such doses are lacking in psychedelic medicine (Kuypers et al. 2019). From a pharmacological standpoint, microdoses are defined as 1% of the pharmacologically active dose (i.e., hallucinogenic dose) (Garner and Lappin 2006; Rani and Naidu 2008). Yet, within the psychedelic science realm, “microdosing” refers to the administration of doses equal to approximately 10% of the hallucinogenic doses that are commonly used recreationally (Fadiman 2011). In either definition, “microdosing” refers to a dose that does not produce hallucinogenic effects. Alternatively, Holze and colleagues (2021) refer to microdoses, minidoses, and psychedelic doses in their LSD-studies (Holze et al. 2021). Because of the inconsistent use of the term “microdosing,” we will refer in this review to low doses of psychedelics (i.e., no or very little hallucinogenic experience) and high doses of psychedelics (i.e. full hallucinogenic experience) in order to avoid confusion. Also, as Ona and Bousa (2020) discuss, “microdosing” does not mean that low doses of psychedelics produce no detectable effects. Hallucinogen and mystical effects may be absent, but these low doses may produce a variety of other effects, such as mood-enhancing and creative effects that can be perceived by the user (Kuypers et al. 2019; Ona and Bouso 2020).

## The antidepressant action of psychedelics

### Antidepressant effects in humans

Over the last decade, there have been a series of clinical trials evaluating the efficacy of psychedelics for treatment of major depressive disorder (MDD) and treatment resistant depression (TRD) (Table 1). The results of these trials are summarized in multiple systematic reviews and meta-analysis and thus will be discussed only briefly in this review (Andersen et al. 2021; Galvao-Coelho et al. 2021; Kuypers 2021; Li et al. 2022).The efficacy of psilocybin in the form of psychedelic-assisted psychotherapy (PAP) has been evaluated under several clinical trial types, including open label and double blind crossover, and has shown promise at reducing depressive symptoms across all clinical trials (Table 1; for a review see (Kuypers 2021). Overall, PAP with high hallucinogenic doses (10–30 mg/70 kg) of psilocybin given once or twice on two separate sessions showed remarkable antidepressant effects. These effects were fast acting and long-lasting, maintained up to 12 months after administration in some studies (Carhart-Harris et al. 2018, 2016; Davis et al. 2021; Griffiths et al. 2016; Gukasyan et al. 2022; Ross et al. 2016) One trial in MDD patients compared the efficacy of PAP with psilocybin with the selective serotonin reuptake inhibitor escitalopram, and although it did not show superiority on the primary endpoint (i.e., 16-item Quick Inventory of Depressive Symptomatology–Self-Report; QIDS-SR-16), it was more effective on secondary outcomes and better tolerated (Carhart-Harris et al. 2021). In fact, in most of the studies mentioned above, psilocybin was well tolerated with minimal adverse effects or events being reported. Collectively, these data suggest that psilocybin produces rapid and sustained antidepressant effects in various depressive populations.

**Table 1:** Antidepressant and hallucinogenic effects of psilocybin and ayahuasca in patients with major depressive disorder or treatment resistant depression

Drug	Type of trial	Participants (age range); Sample size	Dose tested	Physiological response (% or duration of effect)	Hallucinogenic or subjective effects (Duration of effect)	Effective for MDD Duration (% meet response criteria)	Citation
Psilocybin	Open label	MDD (30–57); *N* = 12 (6 F)	10 and 25 mg; 25 mg was administered 1 week after 10 mg. All data was collected after 25 mg	Headache (33%)	–	**HAM-D**: **↓** scores at week 1 **MADRS**: **↓** scores at week 1 **QIDS**: **↓** scores at week 1, 2, 3, 5, and 3 months **BDI**: **↓** scores at week 1 (67%) **↓** scores 3 months (50%)	(Carhart-Harris et al. 2016)
	Open label	TRD; *N* = 19	10 and 25 mg; 25 mg was administered 1 week after 10 mg. All data was collected after 25 mg	–	–	**QIDS**: **↓** scores at week 1 (65%) **↓** scores at week 5 (47%)	(Carhart-Harris et al. 2018)
	Randomized, waiting list (delayed) controlled; clinical trial; 8 week assisted therapy	MDD; *N* = 24 (16 F)	20 mg/70 kg (session 1) and 30 mg/70 kg (session 2)	No adverse effects reported	**VAS**: Drug effect: ↑ Distance from reality: ↑	**GRID-HAM-D**: **↓** scores at week 1 (67%) **↓** scores at week 4 (71%) **↓** scores at 12 month (50%) *scores were not reduced in the delayed group until after psilocybin administration	(Davis et al. 2020; Gukasyan et al. 2022)
	Double-blind cross over design, 2 sessions; very low dose psilocybin controlled	Patients with potentially life-threatening cancer and anxiety and/or mood symptoms; *N* = 51 (25 F)	Low dose: 1 mg/70 kg, High dose: 22 mg/70 kg	Vomiting/nausea (15%) Heart rate: ↑ (60–240 min) Blood pressure: ↑ (60–240 min)	**VAS**: Drug Effect: ↑ (30–360 min) **HRS**: ↑ all six HRS scales **5D-ASD**: ↑ on all dimensions *1 mg/70 kg dose did not produce a significant change on any scales	**GRID-HAM-D**: **↓** scores at week 5 (~ 88%) **↓** scores at 6 months (79%) **BDI:** **↓** scores at week 5 and 6 months *1 mg/70 kg dose did not produce a significant change on any scales	(Griffiths et al. 2016)
	Double-blind cross over design, 2 sessions; active control (niacin)—assisted therapy	Patients with potentially life-threatening cancer and anxiety and/or mood symptoms (22–75); *N* = 29 (18 F)	21 mg/70 kg psilocybin or 250 mg niacin	No adverse effects reported Heart rate: ↑ (90–170 min) Blood Pressure: ↑ (60–180 min)	–	**HADS-D** **↓** scores at day 1 (~ 78%) **↓** scores at week 6 (~ 67%) **↓** scores at 6 months (~ 80%) **BDI**: **↓** scores at day 1 (~ 83%) **↓** scores at week 6 (~ 81%) **↓** scores at 6 months (~ 81%)	(Ross et al. 2016)
	Double-blind, randomized, controlled trial	Patients with moderate-to-severe MDD (21–64); *N* = 59 (20 F)	Two separate doses of 25 mg of psilocybin (or 1 mg psilocybin in the escitalopram group) 3 weeks apart or 6 weeks of daily oral escitalopram (or placebo in the psilocybin group)			**QIDS**: **↓** from the day before to the day after dosing-day 1 **↓** at day 21 (70% psilocybin vs 48% escitalopram)	(Carhart-Harris et al. 2021)
Ayahuasca	Open label	MDD (28–61); *N* = 6 (4 F)	1.76 mg/kg of *N*,*N*-DMT	Vomiting (50%); Heart rate: No change Blood Pressure: No change	–	**HAM-D**: **↓** scores at day 1, 7, and 21 (not at d14) **MADRS**: **↓** scores at day 1, 7, and 21 (not at d14) **BPRS**: No significant change	(Osório Fde et al. 2015)
	Open label	MDD; *N* = 17 (14 F)	1.76 mg/kg of *N*,*N*-DMT	Vomiting (47%); Heart rate: No change Blood Pressure: No change	**CADSS**: ↑ (40–80 min) **BPRS**:	**HAM-D**: **↓** scores at 80–180 min, day 1, 7, 14, and 21 **MADRS**: **↓** scores at 80–180 min, day 1, 7, 14, and 21 **BPRS**: **↓** scores at 40–180 min, day 1, 7, and 21	(Sanches et al. 2016)
	Between subject, double-blind; active placebo (zinc sulfate)	Unipolar MDD (18–60); *N* = 29 (21 F)	0 or 0.36 mg/kg of N,N-DMT	Nausea (71%) Vomiting (57%) Headache (42%)	**HRS:** ↑ in perception, somaesthesia, cognition, intensity and volition (5 of 6 subscales) **CADSS**: No significant changes	**HAM-D**: **↓** scores at day 7 (57%) **MADRS**: **↓** scores at day 1 (50%) **↓** scores at day 2 (77%) **↓** scores at day 7 (64%) **BPRS**: No significant change	(Palhano-Fontes et al. 2019)

↑ = significant increase in scores

**↓** = significant decrease in scores

– = not measures or reported

F = Female

Major depressive disorder (MDD); Treatment resistant depression (TRD); Hamilton Depression Rating score (HAM-D); Montgomery–Åsberg Depression Rating Scale (MADRS); Quick Inventory of Depressive Symptoms (QIDS); Beck Depression Inventory (BDI); Hospital anxiety and depression scale-Depression (HADS-D); Visual Analog Scales (VAS); Hallucinogenic Rating Scale (HRS); 5 Dimensions of Altered States of Consciousness Questionnaire (5D-ASC); Clinician-Administered Dissociative State Score (CADSS); Brief Psychiatric Rating Scale (BPRS)

^*^There are no clinical trials published on the antidepressant effects of LSD

The antidepressant effects of ayahuasca have been evaluated in open label and double-blind active placebo clinical studies. As opposed to psilocybin, ayahuasca was always administered without psychological interventions in these trials, and no preparatory sessions occurred prior to the administration day. Ayahuasca (1.76 mg/kg DMT) produced rapid and sustained antidepressant effects in a small population of MDD patients across two open label clinical trials (Osório Fde et al. 2015; Sanches et al. 2016; Zeifman et al. 2019, 2021). These studies found that ayahuasca produced a significant reduction in depressive symptoms as quickly as 180 min after drug intake and which lasted up to 21 days (response rates were not provided). In a between-subjects, double-blind, active placebo clinical trial, ayahuasca (0.36 mg/kg DMT) produced antidepressant effects from days one to seven (Palhano-Fontes et al., 2019). However, the ayahuasca and placebo groups had similar response rates on day one and two, but not on day seven, making it difficult to fully understand the efficacy of ayahuasca treatment. Importantly, research with ayahuasca is now moving toward a form of DMT which can be delivered in a pill form containing defined quantities of specific β-carbolines (also known as pharmahuasca), which would reduce the confounding of heterogeneous preparations of the brew.

### Proposed model of antidepressant action

**Fig. 2:**
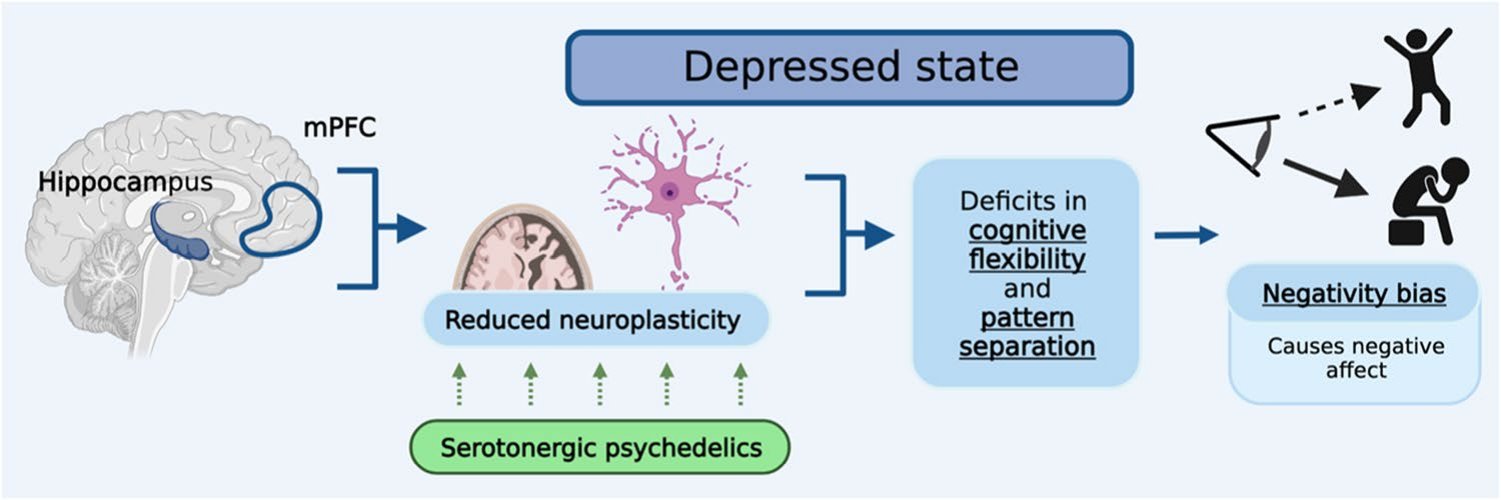
Proposed model of antidepressant action of psychedelics. Depressive disorders are characterized by reduced integrity, function, and connectivity of brain regions that are important for matching mood and goal-directed behavior to a given context (i.e., cognitive flexibility), including the hippocampus and the medial prefrontal cortex (mPFC). Through a direct (e.g., neurotrophic pathways) and indirect (e.g., neuroinflammatory pathways) stimulation of the cellular and molecular mechanisms that underlie neuroplasticity, psychedelics are able to restore the cognitive impairments in attentional set-shifting and pattern separation in a dose- and time-dependent manner. This creates a window of increased cognitive flexibility in which patients can learn to overcome the *negativity bias* that is responsible for the persistent negative affect though the creation of novel cognitive behavioral schemas

The mechanism by which psychedelics exert their presumed antidepressant effects remains elusive. Using a Research Domain Criteria (RDoC) framework, we extended previously proposed models by linking concepts from cognitive neuroscience (negativity bias) with biological mechanisms (neuroplasticity) (Magaraggia et al. 2021) (Fig. 2). According to Aaron Beck’s cognitive model of depression (1963), depressive disorders are often characterized by negative cognitive schemas that lead to a higher tendency of patients to focus on cues possessing negative valence (e.g., depressive rumination) (Beck 1963). This so-called *negativity bias* has been associated with the persistent negative affect and cognitive rigidity of depressed patients (Gollan et al. 2016). It has been proposed that the psychedelic experience allows for an acute disruption of the negative cognitive schemas present in depression through relaxation of a priori beliefs that one might have about the self, the others, and the world (Carhart-Harris and Friston 2019). Subsequently, psychedelics appear to increase long-term psychological and cognitive flexibility, which may open a window of plasticity that facilitates the integration of novel cognitive-behavioral schemas in depressed patients, allowing them to overcome their negativity bias and improve their depressive symptomatology (Davis et al. 2020; Watts and Luoma 2020).

In line with the neurotrophic and neuroinflammation models of depression (Jaggar et al. 2019; Levy et al. 2018; Rhie et al. 2020; Troubat et al. 2021), it has been hypothesized that the underlying biological mechanism involved in the long-term antidepressant effects involve a restoration of neuroplasticity deficits in areas that are important for cognition (Artin et al. 2021; Magaraggia et al. 2021; Vollenweider and Preller 2020) and a decrease in neuroinflammation that is believed to be a causing factor for these deficits (Bouso et al. 2021; Galvao-Coelho et al. 2020). In fact, preliminary in vitro and in vivo studies have shown that psychedelics can increase both structural and functional neuroplasticity in the prefrontal cortex (PFC) (Ly et al. 2018; Shao et al. 2021), and stimulate the proliferation, differentiation, and integration of newborn neurons in the dentate gyrus of the hippocampus (Catlow et al. 2016, 2013; Lima da Cruz et al. 2018; Morales-Garcia et al. 2020). Furthermore, ayahuasca’s antidepressant effects in humans correlated with changes in circulating markers of neuroplasticity and neuroinflammation (de Almeida et al. 2019; Galvao-Coelho et al. 2020). However, exactly how these changes in neuroplasticity and neuroinflammation translate into antidepressant action is still unknown.

According to our model (Fig. 2), the presumed long-term neuroplastic and inflammatory changes induced by psychedelics lead to a restoration of specific cognitive impairments in MDD that may underlie the *negativity bias*. In fact, the *negativity bias* is characterized by high cognitive rigidity. This means that depressed individuals often show inability to match mood and goal-directed behavior to a given context (Anacker and Hen 2017). This deficit can potentially be observed through relevant measures of cognition that are needed to support cognitive flexibility, including attentional set-shifting (ASS) and pattern separation (PS). ASS is the executive function that allows for the formation, maintenance and shift of attentional sets and is often used as a measure of cognitive flexibility in both rodents and humans (Brown and Tait 2016). Impairments in this function have been associated with disorders that are characterized by a reduced integrity of the medial (mPFC), such stress-related mood disorders and schizophrenia (Heisler et al. 2015). Importantly, ASS performance has been found to influence the quality of cognitive restructuring during cognitive behavioral therapy for depression (Johnco et al. 2014), and early improvements in this function during antidepressant treatment were predictive of therapeutic success in a study including 209 MDD patients (Wagner et al. 2018). PS is a mnemonic process that allows for the discrimination of highly similar contextual information, and is necessary for reversal learning, both in neutral and in fearful situations, thus preventing overgeneralized behavioral responses (Yassa and Stark 2011). Compared to ASS, less is known about the mechanisms underlying PS, yet is seems to be strongly influenced by the rate of adult neurogenesis taking place in the subgranular zone of the hippocampal dentate gyrus, and is reduced in conditions characterized by hippocampal deficits such as depression and schizophrenia (Leal and Yassa 2018). By stimulating these underlying biological mechanisms through, for example, pharmacological manipulations, a restoration of correct PS and ASS can be induced in models of depression and schizophrenia (Sahay et al. 2011; Van Hagen 2020). Based on the assumption that psychedelics stimulate neuroplasticity and neurogenesis in the mPFC and hippocampus respectively, we assume that these effects open a period of plasticity in which increased cognitive flexibility supports the learning and integration of novel cognitive-behavioral schemas, which can then be applied into real life scenario with the help of a restored PS performance (Fig. 2). As initial evidence for this model, it was found that a single dose of psilocybin (1 mg/kg) restored the deficits in PS induced by chronic mild stress in young female rats, and this effect correlated with a reduction in depressive-like symptoms in a forced swim test (FST) (Hibicke and Nichols, 2020). Yet, further research is needed to support our hypothesis.

### The role of the acute hallucinogenic and mystical experience in the antidepressant effects of psychedelics

The role of the acute hallucinogenic and mystical effects in the antidepressant effects of psychedelics is currently a matter of debate. The common view has been that these effects are essential for the antidepressant effects as demonstrated, for example, by three RCTs with psilocybin that show correlations between the intensity of the acute mystical experience and the long-lasting therapeutic effects in depression, anxiety, and nicotine addiction (Yaden and Griffiths 2021). In fact, the subjective experience of the patient, and especially the acute psychedelic and mystical effects induced by these agents, play a fundamental role in the psychotherapeutic framework of PAPs. In fact, PAPs are based on anecdotal evidence from the 1950s that was supported by recent studies, indicating that the psychedelic experience can facilitate psychotherapy by promoting emotional acceptance and mindfulness, strengthening the therapeutic “alliance” between the patient and the therapist, and supporting the creation of psychotherapeutic meaning through, for example, an enhanced suggestibility of the patient (for a comprehensive review see Nayak and Johnson (2021).

Contrary to this idea, it was recently suggested that the antidepressant and acute subjective effects of psychedelics may be independent from one another (Olson 2021). This alternative view leans on the assumption that stimulation of neuroplasticity underlies the therapeutic effects of antidepressant drugs, as recently demonstrated for the *N*-methyl-d-aspartate (NMDA) receptor antagonist ketamine. In fact, ketamine produced rapid and sustained antidepressant effects across several open-label, single- and double-blinded clinical studies (Hillhouse and Porter 2015), and it is very likely that these effects are mediated by a stimulation of cortical neuroplasticity (Aleksandrova and Phillips 2021). However, whether its therapeutic effects require hallucinogenic/dissociative effects is unclear. On the one hand, a secondary analysis of previously published RCTs found that, similarly to psilocybin, the dissociative (or hallucinogenic) effects of ketamine were *positively correlated* with the antidepressant effects observed on day seven and were considered the best predictor for antidepressant effects in patients (Luckenbaugh et al. 2014). Yet, other studies have rejected the idea that these effects are important for therapeutic success. In fact, three studies have shown that when ketamine is administered intra-operatively during general anesthesia, while patients are unconscious and unaware of ketamine’s dissociative and hallucinogenic effects, the rapid acting antidepressant effects are still maintained (Jiang et al. 2016; Kudoh et al. 2002; Xu et al. 2017). These results suggest that the antidepressant effects of ketamine may not require an acute mystical experience.

Because of similarities between the neuroplastic effects of psychedelics and ketamine (Aleksandrova and Phillips 2021; Kadriu et al. 2021; Ly et al. 2018), it has been hypothesized that the antidepressant effects of psychedelics are, similarly to ketamine, independent from their psychedelic and mystical properties (Olson 2021). This is an important question, because of the scalability problem of PAPs in the general population that results from the close psychological support that is often required during the acute drug experience that dramatically increases the treatment costs associates to it. However, studies that looked at the effects of intraoperative administration of psychedelics in MDD patients are lacking. Yet, there are other ways by which the role of the acute hallucinogenic and mystical experience can be tested. In fact, there is some evidence showing that sub-chronic administration of low doses of psychedelics that do not induce psychedelic or mystical effects (i.e., “microdosing”) can improve mood and cognition and potentially stimulate neuroplasticity (Hutten et al. 2020; Kuypers et al. 2019; Polito and Stevenson 2019), and may therefore represent an alternative treatment option for depression compared to current psychedelic-assisted therapies (Kuypers 2020). From a pharmacological viewpoint, this hypothesis would translate in the 50% of the effective dose (ED50) for the antidepressant (and neuroplastic) effects being lower than the ED50 for the psychedelic and mystical effects, i.e. that low doses that do not induce psychedelic effects might already be effective at treating depression (graphical abstract, Fig. 1). The next section of this review will examine this hypothesis by discussing the dose-time-dependent effects of psychedelics in both humans and rodents.

## The dose-dependent effects of psychedelics in humans and rodents

**Fig. 3:**
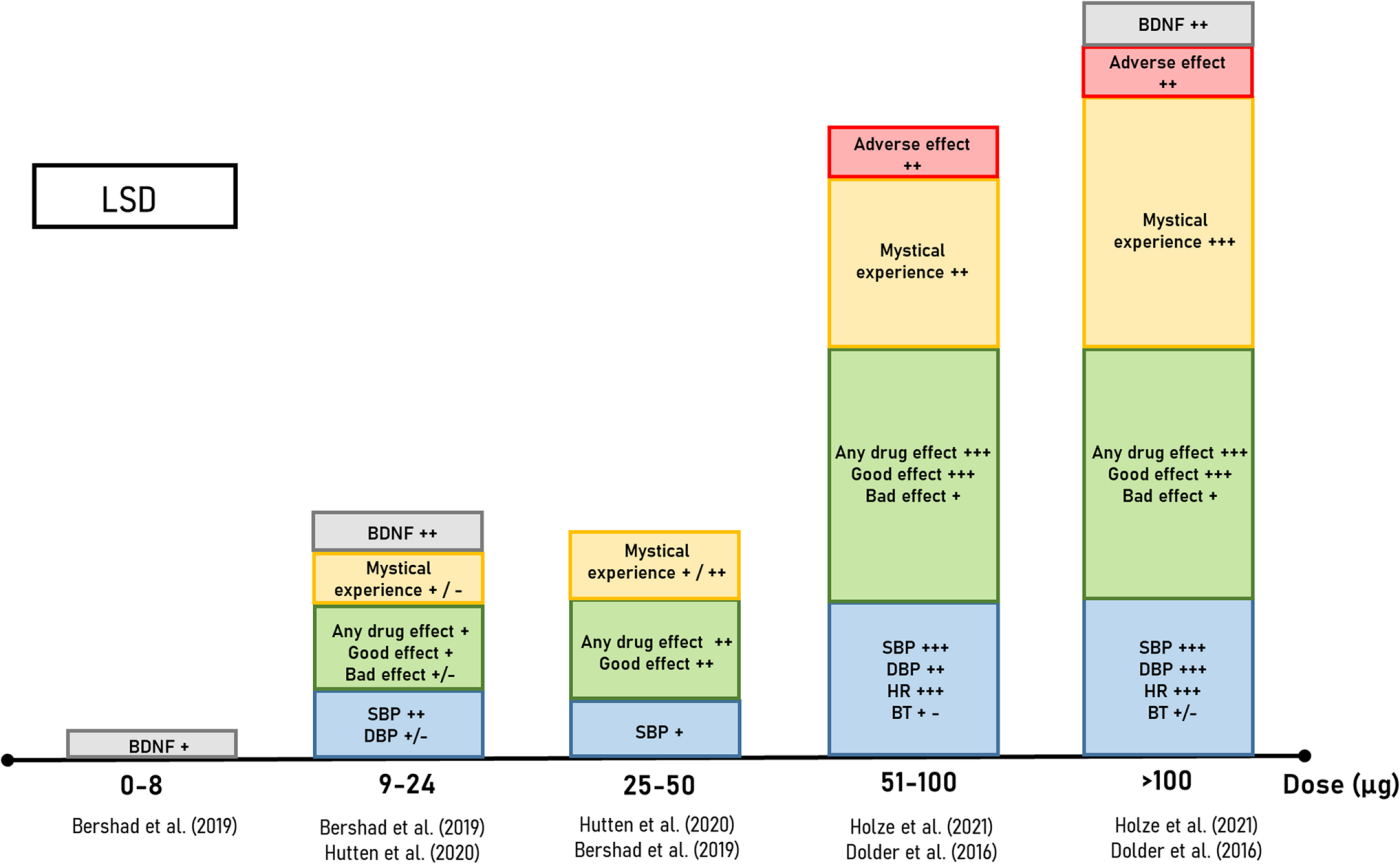
Average estimated effect sizes for LSD. Categories include sympathomimetic effects on heart rate, blood pressure, and body temperature (blue), subjective effects as measured using visual analogue scales for *any*, *good*, and *bad drug effects* (green), mystical experience as measured using the 5 dimensional Altered States of Consciousness scale (yellow), overall adverse effects (red), and blood concentrations of various biological parameters (grey). The relative effect sizes for each category are depicted as large (+ + +), medium (+ +), and small ( +). Contradicting results or insufficient data to estimate an effect size is depicted as + **/ − **. The boxes represent merely a qualitative rather than a quantitative representation of the effect sizes as determined from the information available in each study. LSD tartrate doses have been converted to their bioequivalent base from to facilitate a more direct comparison between studies. *BDNF* brain-derived neurotrophic factor, *BT* body temperature, *DBP* diastolic blood pressure, *HR* heart rate, *SBP* systolic blood pressure

**Fig. 4:**
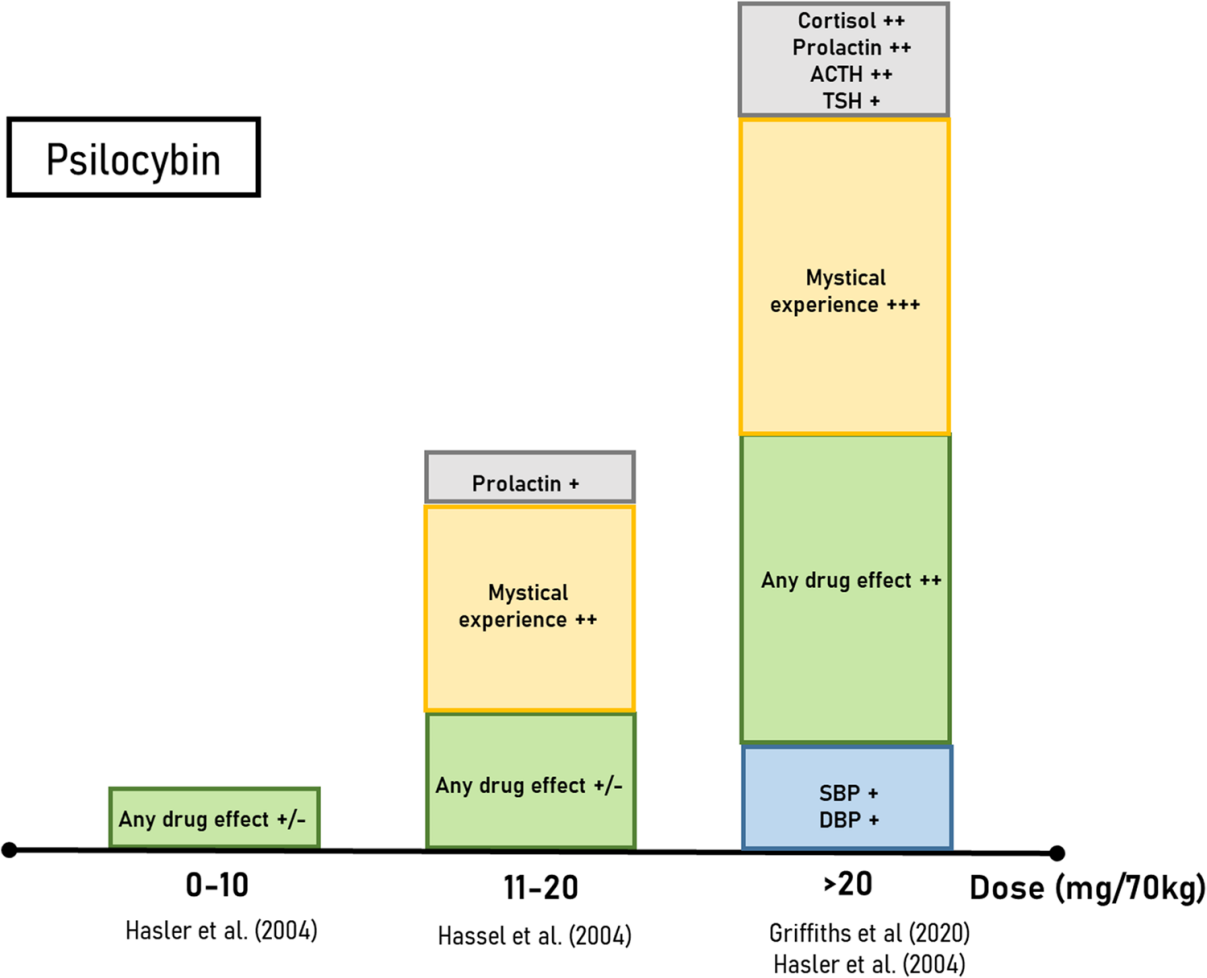
Average estimated effect sizes for psilocybin. Categories include; sympathomimetic effects on blood pressure (blue), subjective effects as measured using visual analogue scales for *any drug effects* (green), mystical experience as measured using the 5 dimensional Altered States of Consciousness scale (yellow) and blood concentrations of various biological parameters (grey). The relative effect sizes for each category are depicted as large (+ + +), medium (+ +), and small ( +). Contradicting results or insufficient data to estimate an effect size is depicted as + **/ − **. The boxes represent merely a qualitative rather than a quantitative representation of the effect sizes as determined from the information available in each study. *ACTH* adrenocorticotropic-releasing hormone, *DBP* diastolic blood pressure, *SBP* systolic blood pressure, *TSH* thyroid stimulating hormone

**Fig. 5:**
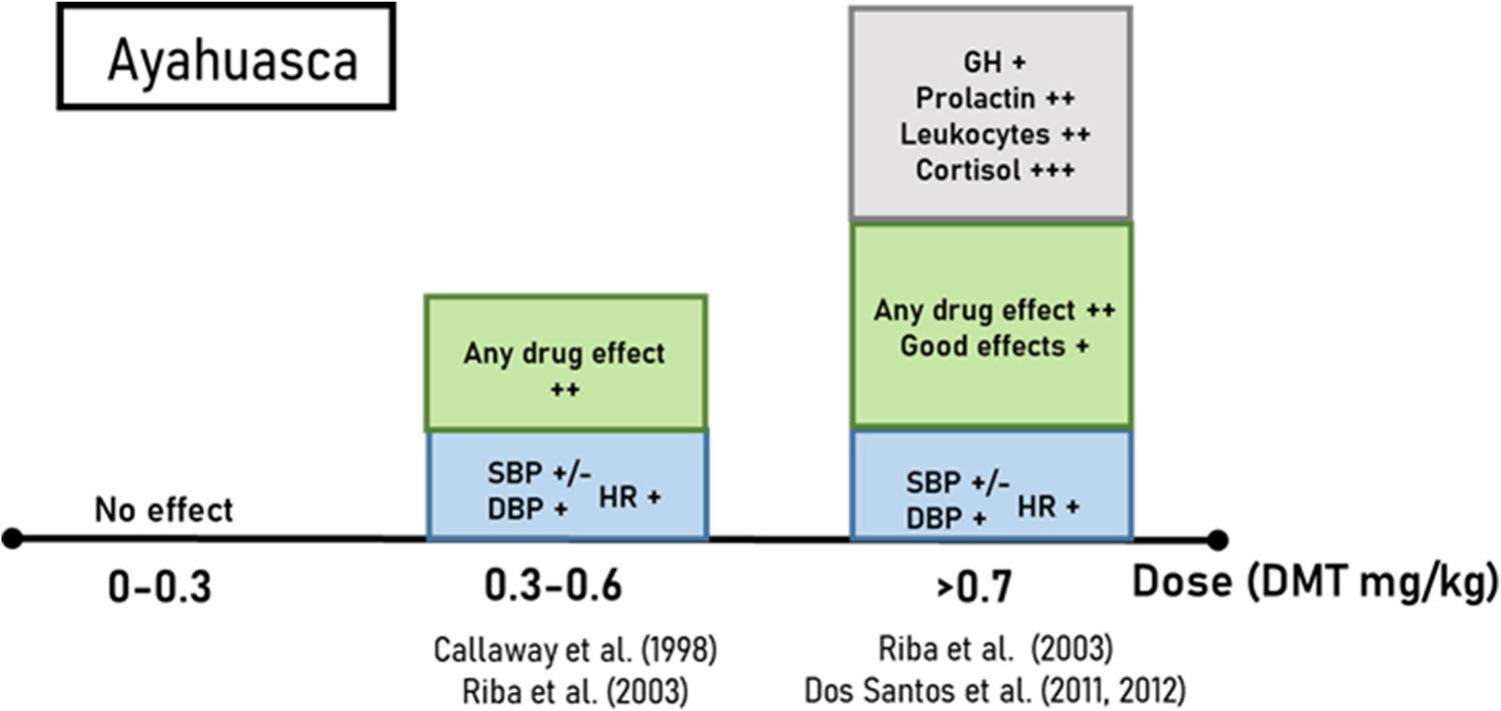
Average estimated effect sizes for ayahuasca. Categories include sympathomimetic effects on heart rate and blood pressure (blue), subjective effects as measured using visual analogue scales for *any* and *good drug effects* (green), and blood concentrations of various biological parameters (grey). The relative effect sizes for each category are depicted as large (+ + +), medium (+ +), and small ( +). Contradicting results or insufficient data to estimate an effect size is depicted as + **/ − **. The boxes represent merely a qualitative rather than a quantitative representation of the effect sizes as determined from the information available in each study. *DBP* diastolic blood pressure, *HR* heart rate, *GH* growth hormone, *SBP* systolic blood pressure

To assess whether the role of the psychedelic and mystical experience is critical for the antidepressant effects of psychedelics, a good understanding of their pharmacokinetic (PK) and pharmacodynamic (PD) relationships is important. This section briefly summarizes the main results of studies that have investigated the various effects of LSD, psilocybin, and ayahuasca*/*DMT, with the aim of identifying dose–response relationships. We included subjective, physiological, and cognitive effects because of their relevance in our hypothesis. For the physiological category, we particularly focused on the sympathomimetic, endocrine and inflammatory effects, since these factors seem to play an important role in the antidepressant effects of psychedelics, as discussed previously. The sympathomimetic effects are particularly interesting for the drug discrimination studies and will be discussed in detail in the next section in which we propose our translational model. The relevance of discussing the dose-dependent effects of psychedelics on subjective and cognitive effects has been discussed in the previous section but will furthermore be discussed in greater depth later on. For translational purposes, human findings will be compared to available rodent data, in order to assess their translatability. The human findings are listed in Table 2 and represented visually in Figs. 3, 4, and 5.

**Table 2:** Hallucinogenic effects of LSD, psilocybin and ayahuasca in healthy controls

Drug	Type of trial	Participants (age range); sample size	Dose tested	Physiological response (duration of effect)	Hallucinogenic or subjective effects (duration of effect)	Citation
LSD	Double-blind cross over design, 4 sessions; placebo controlled (non-active)	Healthy (18–40); *N* = 20 (12 F)	0, 6.5, 13, 26 µg (tartrate)	Heart rate: No change Blood pressure: ↑ 13 and 26 µg (120–180 min) Body temperature: No change	**VAS**: Drug effect: ↑ 13 and 26 µg (120–270 min) Feel high: ↑ 26 µg (120–210 min) Like drug: ↑ 26 µg (120–210 min) Dislike drug: ↑ 26 µg (210 min) Want more: no change **DEQ:** No change **5D-ASC:** ↑ Experience of Unity, Blissful State, and Impaired Control and Cognition **ARCI:** LSD: ↑ 26 µg (120–210 min) No change on other subscales	(Bershad et al. 2019)
	Double-blind, between-subject design; placebo controlled (non-active)	Healthy older adults (mean age = 62.3); *N* = 48 (21 F)	0, 5, 10, 20 µg (tartrate); repeated dosing (6 doses, 4 days apart)	–	**VAS (acute)**: Drug Effect: no change Feel High: no change Unusual thoughts: no change Perceptual distortions: no change Concentration: no change	(Family et al. 2020; Yanakieva et al. 2019)
	Double-blind, randomized; placebo controlled	Healthy; *N* = 24 (12 F)	0, 5, 10, and 20 µg (base)	Heart rate: No change Blood pressure: ↑ 10 and 20 µg (30 min–6 h)	**VAS**: Drug effect: ↑ 10 and 20 µg (60 min–5 h) Good drug effect: ↑ 10 and 20 µg (60 min–5 h) Bad drug effect: ↑ 20 µg (2–5 h)	(Holze et al. 2021; Ramaekers et al. 2021)
	Single-blind cross over design, 2 sessions; placebo controlled (non-active)	Healthy (22–47); *N* = 20 (4 F)	0 and 75 µg (IV)	–	**ASC**: ↑ all 11 dimensions **PSI**: Delusional Thinking: ↑ Perceptual Distortion: ↑ Cognitive Disorganization: ↑ Mania: ↑	(Carhart-Harris et al. 2016)
	Double-blind cross over design, 2 sessions; placebo controlled (non-active)	Healthy (25–60); Study 1: *N* = 24 (12 F) Study 2: *N* = 16 (8 F)	Study 1: 0 and 100 µg (hydrate) Study 2: 0 and 200 µg (hydrate)	Heart rate: ↑ (30 min–10 h) Blood pressure: ↑ (30 min–10 h) Body temperature: ↑ (30 min–10 h) Pupil size: ↑ (30 min–10 h)	**VAS**: Drug effect: ↑ (30 min–16 h) Good drug effect: ↑ (30 min–16 h) Bad drug effect: ↑ 200 µg (30 min–6 h) Stimulated: ↑ 200 µg (30 min–6 h) Happy, open, closeness to other, trust: ↑ (30 min–12 h) **5D-ASC:** ↑ 200 µg on all dimensions **ARCI**: Amphetamine: ↑ 200 µg Morphine-Benzedrine: ↑ 200 µg Pentobarbital-chlorpromazine-alcohol: ↑ 200 µg LSD: ↑ 200 µg	(Dolder et al. 2016)
	Double-blind placebo controlled, cross-over	Healthy (*N* = 16) (8F)	0, 25, 50 100, 200 µg, 200 µg (base) + 40 mg Ketanserin	Heart rate: ↑ 100 and 200 µg (blocked by ketanserin) Blood pressure: ↑ 50, 100, and 200 µg (blocked by ketanserin) Blood mBDNF levels: ↑ 200 µg (blocked by ketanserin)	**VAS**: Drug effect: ↑ 25, 50 100, and 200 µg Good drug effect: ↑ 25, 50, 100, and 200 µg Bad drug effect: ↑ 100 and 200 µg Ego dissolution: ↑ 50, 100, and 200 µg	(Holze et al. 2021)
Psilocybin	Double-blind cross over design, 2–3 sessions; active placebo (methylphenidate)	Healthy (24–64); *N* = 36 (22 F)	30 mg/70 kg Psilocybin or 40 mg/70 kg methylphenidate	Heart rate: ↑ (30 min–3 h) Blood Pressure: ↑ (30 min–6 h)	**VAS**: Drug effect: ↑ (60 min–6 h) **ARCI**: Amphetamine: ↑ Pentobarbital-chlorpromazine-alcohol: ↑ LSD: ↑ **HRS**: ↑ all six scales	(Griffiths et al. 2006)
	Double-blind cross over design, 5 sessions; placebo controlled (non-active)	Healthy (22–44); *N* = 8 (4 F)	0, 3.15, 8.05, 15.05, and 22.05 mg/70 kg	Heart rate: ↑ 22.05 mg/70 kg (60 min) Blood pressure: ↑ 22.05 mg/70 kg (90 min)	**Psychoactive effect**: ↑ All doses (40–120 min) **5D-ASC**: ↑ Dose dependent increased on all scales 8.05 mg/70 kg ↑ oceanic boundlessness 3.15 mg/70 kg no change	(Hasler et al. 2004)
	Double-blind cross over design, 4 sessions; placebo controlled (non-active)	Healthy; *N* = 17 (6 F)	Placebo + placebo Placebo + 15.05 mg/70 kg Psilocybin; 50 mg ketanserin + Placebo; 50 mg ketanserin + 15.05 mg/70 kg psilocybin	–	**5D-ASC**: ↑ Placebo + 15.05 mg/70 kg psilocybin on all scales Pretreatment with ketanserin blocked the effects of psilocybin on all scales	(Kometer et al. 2012)
	Double-blind cross over design, 2 sessions; placebo controlled (non-active)	Healthy; *N* = 25 (9 F)	0 and 11.20 mg/70 kg	–	**5D-ASC:** ↑ all measures except for spiritual experience and anxiety	(Kraehenmann et al. 2015)
Ayahuasca or DMT	Open label	Healthy (26–48); *N* = 15 (All male)	0.48 mg/kg (mean of 35.5 mg)	Heart rate: ↑ (40–180 min) ↓ (120–240 min) Blood pressure: ↑ (20 min) Body temperature: ↑ (120–240 min) Pupil size: ↑ (40–240 min)	**HRS:** ↑ all six HRS scales	(Callaway et al. 1999)
	Double-blind cross over design, 4 sessions; active control (lactose)	Healthy (19–38); *N* = 18 (3 F)	0, 39.8, and 57.4 mg	Heart rate: No change Blood pressure: ↑ 57.4 mg (15–75 min)	**VAS**: Drug effect: ↑ (30 min–4 h) Good drug effect: ↑ (1–3.5 h) Feel high: ↑ (1–4 h) Like drug: ↑ (1–3.5 h) **ARCI**: Amphetamine: ↑ (both doses) Morphine-Benzedrine: ↑ (57.4 mg) LSD: ↑ (both doses) **HRS**: ↑ all six HRS scales	(Riba et al. 2003)
	Double-blind cross over design, 3 sessions; active control (lactose)	Healthy (20–38); *N* = 10 (All male)	Placebo, 1 mg/kg DMT, and 20 mg d-amphetamine	Body temperature: no change	**ARCI**: Amphetamine: ↑ Morphine-Benzedrine: ↑ LSD: ↑ **HRS**: ↑ all six HRS scales	(Dos Santos et al. 2011)
	Double-blind cross over design; placebo controlled (non-active)	Healthy (24–41); *N* = 9 (M)	0.75 mg/kg DMT (placebo followed by one single dose, or 2 repeated doses; 4 h apart)	Heart rate: ↑ (single and repeated) Blood pressure: ↑ (single and repeated) Body temperature: no change	**VAS:** Any effect: ↑ (single and repeated) Good drug effects: ↑ (single and repeated) Bad drug effects: ↑ (repeated only) Drug liking: ↑ (single and repeated) Stimulated: ↑ (single and repeated) High: ↑ (single and repeated) **ARCI**: Amphetamine: ↑ (single and repeated) Benzedrine: ↑ (single only) morphine–Benzedrine: ↑ (single and repeated) LSD: ↑ (repeated only) **HRS**: ↑ all six HRS scales	(Dos Santos et al. 2012)

↑ = significant increase in scores

**↓** = significant decrease in scores

– = not measured or reported

F = Female

Visual Analog Scales (VAS); 5 Dimensions of Altered States of Consciousness Questionnaire (5D-ASC); Addiction Research Center Inventory (ARCI); Psychotomimetic States Inventory (PSI); Hallucinogenic Rating Scale (HRS); Drug Effect Questionnaire (DEQ); Positive and Negative Affect Scale (PANAS)

### Subjective effects of psychedelics

Psychedelics are known for their strong, dose-dependent, subjective effects that span from subtle perceptual changes to a full psychedelic and mystical experiences, with the latter including strong visual hallucinations and ego dissolution. Whereas the first effect is self-explanatory and is shared by other non-serotonergic hallucinogens, the second refers to alterations in the subjective experience of one’s “self” and is a key hallmark of the subjective experience induced by serotonergic psychedelics (Nour et al. 2016). These effects are often measured using either visual analogue scales (VAS) or validated questionnaires, such as the 5-Dimensional Altered States of Consciousness (5D-ASC) scale, the hallucinogenic rating scale (HRS), and the 30-item mystical experiences questionnaire (MEQ30) (Barrett et al. 2015; Riba et al. 2001; Studerus et al. 2011). In fact, administration of psychedelics in healthy volunteers induce acute dose-dependent increases in the scores of these questionnaires (Table 2) (Blasi et al. 2015; Callaway et al. 1999; Dolder et al. 2016, 2017; Dos Santos et al. 2012; Griffiths et al. 2006; Hasler et al. 2004; Kometer et al. 2012; Kraehenmann et al. 2015; Riba et al. 2003; Schmid et al. 2015). Importantly, perceptual changes seem to appear at lower doses compared to the mystical effects. For example, a thorough dose–response study from Holze et al. (2021) showed that 25 µg of LSD is already sufficient to increase VAS scores for *any drug effects*, *good drug effects*, and *drug liking*, but that higher doses (50 µg and higher) are needed to observe the effects on the 5D-ASC and MEQ30 (Fig. 3). Similar results were observed for psilocybin and ayahuasca (Figs. 4 and 5). For example, an oral psilocybin dose of 3.15 mg/70 kg was reported as being psychoactive, but did not increase ratings on any of the 5D-ASC subscales (Hasler et al. 2004). Moreover, the effects of psychedelics on specific dimensions of the 5D-ASC and MEQ30 also seem to be dose-dependent. High doses of psilocybin (11.20–30 mg/70 kg) increased ratings on most dimensions of the 5D-ASC, whereas treatment with 8.05 mg/70 kg psilocybin only increased ratings on the two subscales *oceanic boundlessness* and *reduction of vigilance* (Hasler et al. 2004). Furthermore, an increase in the *anxious ego dissolution* 5D-ASC subscale appears only at high doses of psychedelics and often together with an increase in VAS scores for *bad drug effects*, as shown by recent studies with LSD and psilocybin (Hasler et al. 2004; Holze et al. 2021).

Importantly, the 5-HT_2A_ receptor seems to be essential for the acute subjective effects of psychedelics as pre-treatment with the antagonist ketanserin, blocked the acute subjective effects of LSD, psilocybin, and ayahuasca in humans (Holze et al. 2021; Preller et al. 2017; Valle et al. 2016; Vollenweider et al. 1998). Moreover, a preliminary positron emission tomography (PET) study in healthy volunteers with the radioligand [11C]Cimbi-36 revealed that the occupancy of this receptor after administration of various doses of psilocybin was predictive of the intensity of these effects (Madsen et al. 2019). In line with previous studies, noticeable perceptual effects were reported after administration of the lowest dose (3.5 mg/70 kg; oral) which led to a receptor occupancy rate of 43%. Yet, because of the limited sample size of this study (*n* = 1 per dose group), further studies are needed to confirm these results (Hasler et al. 2004) (Fig. 4).

### Drug discrimination of psychedelics

A unique tool to measure the subjective effects of drugs is the drug discrimination paradigm. The correlation between discriminative stimulus properties of drugs and their subjective effects in humans has been well documented (Bolin et al. 2016). The procedure requires the organism to discriminate the pharmacological effects of a drug from its absence (i.e., the vehicle condition) and these pharmacological effects reflect subjective effects (as opposed to objective). In addition, the drug is serving as a stimulus, much in the same way that a light or tone might be used as a discriminative stimulus in a learning study. More importantly, human drug discrimination studies have generally confirmed that the subjective effects of drugs in humans correlate with the discriminative stimulus properties of drugs in nonhuman animals. Also, the discriminative stimulus properties of drugs (and therefore their subjective effects) are mediated by specific activity at neurotransmitter receptors in the central nervous system (Balster 1988; Porter et al. 2018). The section below reviews some of the nonhuman drug discrimination studies for LSD, psilocybin, and DMT. Unfortunately, from a translational perspective, there have been no psychedelic drug discrimination studies reported with human subjects.

#### LSD drug discrimination

The discriminative stimulus properties of LSD were studied in the early 1970s, as the drug discrimination field was emerging and the method established as a mainstream behavioral pharmacology assay (see Porter et al. 2018 for early history of drug discrimination). Hirschhorn and Winter (1971) were the first to demonstrate that LSD (0.25 µmol/kg) could be established as discriminative stimuli in rats trained to discriminate LSD from saline. One focus of these early drug discrimination studies was to determine the similarities and differences of the discriminative cues of drugs across different drug classes (Hirschhorn and Winter 1971). For example, Järbe (1980) trained pigeons to discriminate LSD (40 or 50 mg/kg) and found that DMT and psilocybin fully substituted for LSD (replicating previous findings in rats) (Jarbe 1980). However, ∆^9^-THC, morphine, and pentobarbital *did not* produce LSD-appropriate responding. Thus, these early drug discrimination studies supported the idea that hallucinogenic drugs shared discriminative stimulus properties that were unique to this drug class. Given that the discriminative stimulus properties of drugs correlates highly with activity at one or more specific neurotransmitter receptors (Balster 1988; Porter et al. 2018), there was interest in determining the underlying mechanism(s) mediating hallucinogenic drugs. There is a correlation between the potency (ED_50_ values) of these drugs relative to LSD in rats trained to discriminate LSD from saline with the potency (*K*_i_) of these drugs in humans at 5-HT_2A_ and 5-HT_2C_ receptors (see review by Nichols 2016).

However, LSD has also activity at other, non-serotonergic receptors in drug discrimination studies. White and Appel (1982) demonstrated that while LSD and the ergot lisuride share discriminative stimulus properties in rats, LSD’s cue appears to be mediated primarily by serotonin mechanisms; whereas lisuride’s cue was mediated primarily by dopamine mechanisms. Given that both drugs shared discriminative stimulus properties, there obviously are shared underlying receptor mechanisms for these two psychedelic drugs. Based on their findings in this study they concluded that LSD’s psychedelic effects probably depend primarily on serotonergic mechanisms. What White and Appel did not know at that time was that LSD’s effects and underlying mechanisms are time-dependent (White and Appel 1982). Nichols (2016) provides a nice discussion of what he calls a “temporal switch.” With 30 min injection times, the LSD discriminative cue appears to be mediated primarily by activation of 5-HT_2A_ receptors. Antagonist studies with both selective and nonselective antagonists have confirmed the finding that the discriminative stimulus properties of psychedelics appear to be mediated primarily by activation of 5-HT_2A_ receptors (Fiorella et al. 1995; Nielsen et al. 1985; Schreiber et al. 1994). In contrast, when 90 min injections times are used, LSD’s cue (0.08 mg/kg dose used for 30 and 90 min testing) appears to be mediated by activation of dopamine receptors and evidence of roles for both D_2_ and D_4_ receptors have been reported for the delayed onset effects of LSD (Marona-Lewicka and Nichols 2007; Marona-Lewicka et al. 2005). As discussed by Marona-Lewicka et al. (2005) and Nichols (2016), this temporal switch between serotonin and dopamine mechanisms seen in LSD drug discrimination in animals appears to correlate with the effects of LSD in humans as reported by Freedman (1984). He writes that the early phase is characterized by a psychedelic experience followed by a paranoid state (4–6 h after administration) that may be similar to amphetamine-induced psychosis seen in humans. These time-dependent effects on serotonin and dopamine mechanisms may certainly be a factor in the aversive effects that are sometimes reported for psychedelic trips, especially for higher doses (see Fig. 3) (Freedman 1984).

#### Psilocybin drug discrimination

Koerner and Appel (1982) trained 15 rats to discriminate 1.0 mg/kg psilocybin from saline in two-lever task using 30 min sessions. A time course showed that Psilocin and LSD fully substituted for psilocybin at lower doses. The ED_50_ value for psilocybin was 0.24 mg/kg; for psilocin 0.17 mg/kg and for LSD 0.038 mg/kg; the slopes were parallel (Koerner and Appel 1982). Winter et al. (2007) trained rats to discriminate psilocybin (0.5 mg/kg), LSD (0.1 mg/kg), 3,4-methylenedioxymethamphetamine (MDMA) (1.5 mg/kg), or phencyclidine (3.0 mg/kg) from placebo. In the psilocybin-trained rats, both LSD and psilocin fully substituted for psilocybin with results similar to Koerner and Appel (1982). While DMT produced 73% substitution for psilocybin, falling just short of the 80% criterion, [-]-2,5-dimethoxy-4-methylamphetamine (DOM) fully substituted for psilocybin (Winter et al. 2007). Through a series of antagonism tests, their results suggested that stimulus control for psilocybin is partially mediated by 5-HT_2A_ receptors. Specifically, the serotonin 5-HT_2A_ antagonists M100907 and ketanserin partially antagonized the psilocybin discriminative cue. As aforementioned, ketanserin has been shown to block the psychotomimetic effects of psilocybin in humans (Vollenweider et al. 1998).

#### DMT and DMT derivatives drug discrimination

In the late 1970s and early 1980s Richard Glennon and colleagues conducted a series of drug discrimination studies with the methoxylated derivative of DMT, 5-OMe-DMT (aka 5-MeO-DMT, a methoxylated derivative of DMT) examining its discriminative stimulus properties. In one study (Glennon et al. 1980) trained rats to discriminate 1.5 mg/kg 5-OMe-DMT in order to examine the correlation between 5-HT affinity and the discriminative stimulus properties of 5-OMe-DMT. They also tested 13 other hallucinogenic drugs to determine if they would substitute for 5-OMe-DMT’s discriminative cue. There was a correlation of *r* =  − 0.86 (*p* < 0.001) between the ED_50_ values for substitution to the discriminative stimulus cue for 5-OMe DMT and their binding affinity to 5-HT receptors. Glennon et al. (1980) also demonstrated that in rats trained to discriminate either 1.5 mg/kg 5-OMe DMT or 0.096 mg/kg LSD, there was cross-generalization between these two psychedelics and, therefore, concluded that their discriminative stimuli are mediated via a common serotonergic mechanism. More recent studies confirmed that the discriminative stimulus properties of DMT are similar to those of other psychedelic drugs, including LSD, DOM, MDMA, psilocybin, 5-methoxy-DMT, and 2,5-Dimethoxy-4-iodoamphetamine (DOI) (Gatch et al. 2009; Smith et al. 1998; Winter et al. 2007).

While the early studies did not specify which specific serotonergic receptor(s) mediated the discriminative stimulus for 5-OMe-DMT, a couple of studies demonstrated a major role for 5HT_1A_ receptors. In rats trained to discriminate 1.25 mg/kg 5-OMe-DMT, a series of drugs with serotonergic agonist activity was tested, including LSD and various 5-HT_1A_ receptor agonists such as 8-hydroxy-2-(di-n-propylamino) tetralin (8-OH-DPAT). Based on a comparison of the potencies of the test drugs in generalization tests and their binding affinities at 5-HT receptors, the authors concluded that the 5-HT_1A_ receptor subtype appeared to play the most important role in mediating 5-OMe-DMT’s discriminative stimulus (Spencer et al. 1987). Finally, some evidence supports a role for *both* 5-HT_2C_ and metabotropic glutamate receptor 2 (mGluR2) receptors in the discriminative stimulus effects of DMT (Carbonaro et al. 2015). In conclusion, it is clear that the discriminative stimulus for DMT (and 5-OMe-DMT) involves agonism at 5-HT_1A_ receptors, but 5-HT_2A_ agonism also is involved. In addition, interactions at other receptors (e.g., mGluR2/3) may play some role in DMT’s discriminative stimulus properties.

In summary, the pharmacological mechanism of action in drug discrimination models, especially the role of 5-HT_2A_ and 5-HT_1A_ receptors, depends on various factors, including species difference (e.g., LSD has more of a 5HT_1A_ component in the mouse than in the rat; Benneyworth et al. 2005; Winter et al. 2005); drug (e.g., DMT has more of a 5HT_1A_ component in its discriminative stimulus effects than LSD at 30 min injection interval); training dose; Young et al. 1986); and injection interval (e.g., LSD has more of a 5HT_2A_ component in its discriminative stimulus effects at a short injection interval; Marona-Lewicka et al. 2005). In addition, the drug discrimination model may have vertical (i.e., between species) translational validity, as suggested by the reversal of psilocybin’s discriminative stimulus effects by ketanserin in rats and its hallucinogenic effects in humans. Therefore, in addition to the head-twitch response (HTR) model, drug discrimination is an informative in vivo assay for characterization of the hallucinogenic properties of psychedelic drugs.

### Head-twitch response

Measuring the psychedelic experience in rodents is a complicated, if not impossible, task. Yet, there are behavioral models that have demonstrated substantial predictive validity for measuring psychedelics effects in rodents. For example, the HTR in mice is a clear and distinct behavior that nicely correlates with the psychedelic potential of drugs having agonistic activity at the 5-HT_2_ receptor (Halberstadt et al. 2020). The HTR is induced by 5-HT, its precursor, 5-hydroxytryptophan (5-HTP), and 5-HT_2A_ agonists such as mescaline, quipazine, DOI, and DOM (Fozard and Palfreyman 1979; Green et al. 1983). One advantage of this model is that animals do not have to be trained daily for several months, and that drug testing is not limited by the sensitivity of the animals to the behavior-disruptive effects of drugs (Schreiber et al. 1995). However, from a translational perspective, a disadvantage is that this behavioral assay cannot be directly assessed in humans. Using an automated system for registering HTR in C57BL/6 J mice, strong, positive correlations were shown between the potency of psychedelics (including DOI, LSD and DMT; Table 3) to induce HTRs and (1) reported psychedelic potencies in humans (*r* = 0.9448); and (2) published drug discrimination ED50 values for substitution in rats trained with either LSD (*r* = 0.9484) or DOM (*r* = 0.975). The authors concluded that “*the HTR assay also appears to show significant predictive validity*, *confirming its translational relevance for predicting subjective potency of hallucinogens in humans*” (Halberstadt 2015; Halberstadt et al. 2020; Klein et al. 2021). Using the same automated system as mentioned above, studies were performed with psilocybin and 17 tryptamines and all induced HTR in mice, consistent with a LSD-like behavioral profile (Halberstadt et al. 2020). The potency for inducing HTWs was LSD >  > DOI ≥ psilocybin > DMT. Interestingly, the non-hallucinogenic 5-HT_2A_ agonist, lisuride, did not induce head twitches (Halberstadt & Geyer 2013), hence providing an important negative control for this behavioral assay. Also, the fact that hallucinogenic/psychedelic effects are not produced by “flooding” the brain with increased levels of serotonin with drugs like 5-hydroxytryptophan (5-HTP), selective serotonin reuptake inhibitors (SSRI’s), MAOI’s, or tricyclic antidepressants (TCA’s), indicates the importance of individual serotonin receptors in mediating psychedelic/hallucinogenic effects—i.e., only a select subset of 5-HT receptors appear to be responsible for these effects.

**Table 3:** Translational studies with psilocybin in rodent and humans

Effect	Mouse	Rat	Human
Antidepressant (long-term)	Sucrose Preference test: 1 mg/kg, i.p (Hesselgrave et al. 2021) Learned helplessness assay: 1 mg/kg (Shao et al. 2021)	Forced Swim test: 1 mg/kg, i.p (Hibicke & Nichols 2020)	Validated questionnaires: 0.28–0.43 mg/kg, oral (Carhart-Harris et al. 2016; Davis et al. 2020)
Cognitive (long-term)	N.A	Object Pattern Separation: 1 mg/kg, i.p	Convergent and divergent thinking: 0.17 mg/kg, i.p (Mason et al. 2021)
Neuroplastic	Cortical spine and size density: 1 mg/kg (Shao et al. 2021)	N.A	N.A
Neuroinflammatory	N.A	N.A	N.A
Subjective	N.A	ED50 Drug Discrimination substitution Drug stimulus of psilocybin 1 mg/kg, i.p (Koerner & Appel 1982)	Subjective ratings of drug effects: ≥ 0.05 mg/kg, oral (Hasler et al. 2004)

### Physiological effects

Psychedelics have acute, dose-time-dependent, sympathomimetic effects in humans, although some differences in these effects exist between LSD, psilocybin, and ayahuasca. After oral administration of low doses (9 µg) of LSD, there are gradual increases in blood pressure (BP), followed by an increase in heart rate (HR) at medium doses or higher (> 50 µg) (Holze et al. 2021; Ramaekers et al. 2021). Increases in body temperature at high doses (> 100 µg) also have been reported, although results are inconsistent between studies (Dolder et al. 2016; Holze et al. 2021) (Table 2 and Fig. 3). Only high doses of psilocybin (> 20 mg/70 kg) share similar stimulatory effects on BP as LSD and ayahuasca, and no increases in HR have been reported after administration of this drug so far (Griffiths et al. 2006; Hasler et al. 2004) (Tables 1 and 2, Fig. 4). Finally, administration of ayahuasca also gradually increased BP and HR at DMT doses of 0.3 mg/kg or higher (Callaway et al. 1999; Dos Santos et al. 2012; Riba et al. 2003) (Table 2, Fig. 5).

Acute effects of psychedelics also include changes in levels of various physiological parameters, including hormones, markers of the immune system, and growth factors. Studies in healthy volunteers have shown that, compared to placebo, high doses of LSD, psilocybin, and ayahuasca acutely increased plasma levels of circulating hypothalamic–pituitary–adrenal (HPA) axis hormones, including cortisol (Dos Santos et al. 2012; Hasler et al. 2004; Schmid et al. 2015; Strajhar et al. 2016; Strassman and Qualls 1994; Uthaug et al. 2020). Unfortunately, dose–response studies for this effect have been made only for psilocybin and intravenous DMT, but not LSD and ayahuasca*.* Whereas psilocybin’s threshold dose for achieving this effect lies between 22.05 and 15.05 mg/70 kg (Hasler et al. 2004), DMT’s increase in blood cortisol levels was observed only at doses equal to 0.2 mg/kg and higher. Interestingly, LSD-induced (200 µg; oral) changes in plasma cortisol and corticosterone concentrations were related to a positive and stimulant psychedelic experience but not to anxiety (Strajhar et al. 2016). There is evidence to suggest that the stimulatory effect of psychedelics on the HPA axis do not persist after drug excretion, at least for ayahuasca. In fact, the differences in plasma cortisol levels between placebo-treated and ayahuasca-treated subjects disappeared 48 h after administration (Galvao et al. 2018). Besides acting on the HPA system, high doses of psychedelics also seem to affect levels of other circulating hormones, including prolactin, oxytocin, and epinephrine (Dos Santos et al. 2012; Hasler et al. 2004; Schmid et al. 2015; Strajhar et al. 2016; Strassman & Qualls 1994; Uthaug et al. 2020), although there is insufficient data to make meaningful conclusions about these effects.

### Cognitive effects

Publications on the effects of psychedelics on cognition in both healthy and diseased are limited. In healthy volunteers, LSD, psilocybin, and ayahuasca seem to have *acute* detrimental effects on various aspects of cognition in a dose-dependent fashion. For example, hallucinogenic doses of LSD (100 µg, oral) acutely impaired executive functioning and working memory, whereas no effects were observed at doses of 26 µg or lower (Bershad et al. 2019; Hutten et al. 2020; Pokorny et al. 2020; Schmid et al. 2015). Similar to LSD, psilocybin also caused a dose-dependent reduction in associative learning, working memory, episodic memory, visual perception, and psychomotor performance at doses of 10 mg/70 kg and higher (Barrett and Griffiths 2018). Likewise, one study showed a similar detrimental effect on executive functioning of standard ayahuasca doses (Bouso et al. 2013). Interestingly, there is preliminary evidence suggesting potential attention-enhancing effects of low doses of psychedelics. In fact, Hutten et al. (2020) reported significant decreases in attentional lapses in the psychomotor vigilance task (PVT) after administration of 5 and 20 µg of LSD. Importantly, there seems to be a mediation by the 5-HT_2A_ receptor for the cognitive impairing effects of psychedelics, at least for LSD. In fact, the study from Pokorny et al. (2019) showed that LSD’s negative effects on cognition were blocked by pre-treatment with ketanserin (40 mg). Future studies should investigate whether this applies to psilocybin and ayahuasca as well.

Preliminary evidence suggests that psychedelics may positively affect cognition in the long term. A recent randomized, double-blind, placebo-controlled, crossover study showed improvements in visuospatial memory and phonological verbal fluency the day after administration of 50 µg of LSD in healthy volunteers (Wießner et al. 2022). Yet, the study also showed impairments in cognitive flexibility at this time point, as measured by the Wisconsin Card Sorting Task (WCST). The latter finding is in contrast with the positive effects of psychedelics on cognitive flexibility that have been observed in a series of studies. For example, standard ayahuasca doses increased scores in the WCST the day after its administration in healthy volunteers compared to the day prior (Murphy-Beiner and Soar 2020), and similar improvements were observed in regular ayahuasca users one year after baseline assessment (Bouso et al. 2012). Moreover, high doses of ayahuasca and psilocybin influence cognitive thinking style days to weeks after administration (Mason et al. 2021, 2019; Uthaug et al. 2018), and psilocybin (11.9 mg/70 kg) also increased scores of novelty in the Alternative Users Test as a measure of creative thinking at 1-week follow-up compared to placebo (Mason et al. 2021). Importantly, similar effects on cognitive flexibility may occur in depressed populations as well. A recent open-label study by Doss and colleagues (2021) in 24 MDD patients undergoing psilocybin therapy showed an increase in cognitive flexibility that lasted for at least 4 weeks after treatment. However, this data did not correlate with a reduction in depressive symptoms, and more studies are needed to better identify its relationship with the antidepressant effects of psychedelics (Doss et al. 2021).

Other beneficial long-term effects of psychedelics on cognition may be induced upon intermittent low dosing in humans, yet evidence in support of this idea is mostly limited to surveys and netnographic studies. These have reported subjective improvements in various aspects of cognitive functioning, such as attention and memory (Cameron et al. 2020; Hupli et al. 2019; Lea et al. 2020a, 2020b). A prospective study is by Szigeti and colleagues (2021), which adopted a citizen-science self-blinding design to investigate the long-term effect of low doses of psychedelics on cognition using a series of computerized touch-screen tasks. In this study, participants were free to decide the psychedelic substance and dose for a total treatment duration of 4 weeks. The results showed no changes in overall cognitive performance, but slight improvements in local cognitive functioning at week 4 and 9 after the start of the treatment compared to placebo (Szigeti et al. 2021). To further investigate this relationship, a recent double-blind, placebo-controlled study with LSD has showed negligible effects of four repeated doses of the drug (13 or 26 μg administered at 3–4 days intervals) on various aspects of cognition, 4 days after the last dosing session (de Wit et al. 2022). Together, these results indicate that psychedelic “microdosing” may not be very effective improve cognitive functioning, yet further research should focus on specific functions which have shown potential.

As with human studies, there are only a limited number of preclinical studies in rodents that have investigated the effects of psychedelics on cognitive processes. Like in human studies, psychedelics show a mix of both cognitive-enhancing and cognitive-impairment properties in rodents, largely depending on the substance, its dose, and the task used. Moreover, other important factors that determine these effects in rodents are strain, age, and environmental factors. For example, the effects of psilocin (0.2, 0.4, 0.8 mg/kg) were investigated in two strains of mice in the Y-maze light–dark discrimination task, which is a cognitive flexibility task (Castellano 1978). Interestingly, psilocin both *improved* and *impaired* cognitive flexibility for the C57BL/6 J and DBA/2 J mice, respectively (Castellano 1978). This might be explained by the 5-HT_2A_ and 5-HT_2C_ receptors, both of which are targeted by psychedelics, that functionally antagonize each other and have opposing effects on cognitive flexibility in rodents (Amodeo et al. 2020; Boulougouris and Robbins 2010; Meneses 2007).

## A translational framework to study the antidepressant effects of psychedelics

Several conclusions can be drawn from this review of human and rodent LSD, psilocybin, and ayahuasca/DMT data. *First*, all compounds induced physiological and/or subjective findings at doses *below* their therapeutic doses. Although it must be noted that no studies are available that looked at the dose–response curves for the antidepressant effects in humans. *Second*, there is a general paucity of data. Many more studies have been conducted with LSD in healthy humans than with psilocybin, whereas the latter is predominantly used therapeutically in MDD patients, hampering horizontal translation. *Third*, PK/PD relationships have to be performed on plasma exposure data, but most clinical studies did not measure plasma levels. *Fourth*, effects on cognition can differ widely depending on a variety of factors, thus emphasizing the need for a different approach. Finally, this review started with the assumption that psychedelics can be used therapeutically to treat MDD and other depressive disorders. Yet, we urge some caution in the absence of well controlled clinical trials and the many questions to be resolved as to the exact role of the subjective, psychedelic and mystical experience in the antidepressant effects of psychedelics. As Hall and Farrell (2021) recently reviewed, in the 1950s, LSD was not a successful “stand-alone” treatment for the treatment of alcoholism and many mistakes were made. They point out a “*need for caution in the revival of their therapeutic use. Clinical trials of their safety and efficacy need to be conducted in larger*, *more representative patient samples and under conditions more like routine clinical practice*” (Hall and Farrell 2021). Our current review supports this position and highlights the need for such studies.

The findings presented in this review do not allow us to draw any firm conclusions with regard to the role of the psychedelic and mystical experience in the therapeutic effects of psychedelics as measured by differences in the ED50 values of these effects. Yet, preliminary data for the dose-dependent effects is available for psilocybin, and therefore we use this compound as an example of how we aimed to address our research question (Table 3). On the one hand, the ED50s for the discriminative stimulus (Koerner and Appel 1982) and HTR (Halberstadt et al. 2020) effects of psilocybin in rodents have been evaluated, as is the dose–response curve for the psychedelic and mystical effects in healthy human volunteers (Hasler et al. 2004). On the other hand, the effects of psilocybin on depressive symptoms, long-term cognitive flexibility, and neuroplasticity in both rodents and humans mainly have been tested using fixed doses of the drug, with no dose–response curve being established so far. In these studies, positive effects of the drug were observed with doses that are higher than the effective doses for the subjective effects. Yet, vertical translation is often limited by the use of outcome measures with debatable validity (e.g., FST for the antidepressant effects in rodents), or the lack thereof (e.g., no validated human biomarkers of neuroplasticity exist). Moreover, vertical translation is further hampered by the scarcity of PK/PD data in both humans and rodents, such as plasma exposures after drug administration. This data is essential when the goal is to compare the dose–response curves for the therapeutic and adverse events of a drug when different routes of administration have been used across studies (i.p. in rodents vs. oral in humans, see Table 3). It is important to note that the preclinical studies have similar limitations to the clinical studies in that limited dose ranges have been tested. Many of these studies have found that the antidepressant doses in rodents overlap with the discriminative stimulus and HTR doses. Taken together, these data highlight the need for translational studies to investigate these effects in order to determine their role in the therapeutic effects of psychedelics. Here, we propose a translational framework that can address some important issues in this field.

Firstly, we highlight the need for thorough investigations of the PK/PD relationships for the antidepressant effects of psychedelics in both humans and rodents (graphical abstract; Fig. 1). What drug exposures are required to observe these effects? What are the minimal effective exposures? What are the correlations between the therapeutic effects, central and peripheral drug exposures, and target occupancy? Modern neuroimaging techniques, such as PET scans, have been useful tools for this purpose. For example, studies with the antipsychotic haloperidol have shown that the clinical response, hyperprolactinemia, and extrapyramidal side effects are predicted by the magnitude of D_2_ receptor occupancy by the drug as measured by PET using [^11^C]raclopride (Kapur et al. 2000). We believe that similar studies with psychedelics are essential to better understand their antidepressant effects in relation to 5-HT_2A_ receptor occupancy. For example, the aforementioned PET study by Madsen et al. (2019) shows correlations between psilocin plasma levels and 5-HT_2A_ receptor occupancy and the subjective effects of psilocybin. Yet, this study only included healthy volunteers and observations were limited to only one person per treatment condition. We highlight the importance of this work and the need to perform more radioligand displacement studies for targets that seem to be involved in the antidepressant effects of these drugs, including the 5-HT_2A_ and the 5-HT_1^_ receptors. For example, to investigate the correlation between the receptor occupancy by psychedelics and their antidepressant and hallucinogenic effects. Because of the lack of validated markers of neuroplasticity in humans, similar studies should be performed in rodents to further evaluate the PK/PD relationship for the effects of psychedelics on neuroplasticity and neurogenesis. Yet, we also highlight the need for future studies investigating inter-species differences in metabolism and receptor binding affinities of these drugs. For example, there is evidence for the inter-species differences in the binding affinity of the active metabolite psilocin as a result of differences in the amino acid sequence of the 5-HT_2A_ receptor (Almaula et al. 1996; Gallaher et al. 1993; M. P. Johnson et al. 1994). Such findings are essential to allow for a correct vertical translation of past and future findings that is needed to gain fundamental knowledge of how psychedelics work in both healthy and depressed.

Secondly, and linked to our hypothesis, we propose to assess the role of the hallucinogenic and mystical experience in the antidepressant effects of psychedelics by investigating the differences in drug exposures needed to achieve these effects. We argue in favor of the possibility that the ED50 for the antidepressant effects of psychedelics is lower than for the mystical and hallucinogenic effects. Yet, data for the antidepressant effects of psychedelics is limited to high hallucinogenic doses only. For example, RCTs with psilocybin have used only doses that are approximately twice as high as the threshold dose for the mystical effects (8.05 and 11.20 mg/70 kg vs. > 20 mg/70 kg) (Tables 1 and 2). One source of evidence to support our idea are anecdotal studies investigating the effects of intermittent low doses of psychedelics as mood and cognitive enhancers. The aforementioned placebo-controlled citizen science study of Szigeti et al. (2021) shows that sub-chronic administration of low doses of psychedelics may have positive effects on mood and cognition. In this study, LSD at doses equal to 13 ± 5.5 µg were chosen by 61% of the participants as active treatment. The dose-dependent effects of LSD presented in Fig. 3 shows that 13 µg falls in the 9–24 µg bracket where there was no clear effect for “*mystical experience*” observed (“contradicting results or insufficient data to estimate an effect size”) (Bershad et al. 2019; Hutten et al. 2020). This finding is consistent with our idea that the ED50 for the therapeutic effects of psychedelics might be lower than the ED50 for the psychedelic effects. However, these doses are sufficient to produce subtle subjective effects that can be perceived by the user, as demonstrated by 41% of participants of the study of Szigeti et al. (2021) that correctly guessed what treatment they received (see also Figs. 3–5; small effect sizes were found for “*any drug effects*” and “*good effect*” in the 9–24 µg dose bracket). Importantly, the ability of the participants to guess whether they were taking placebo or the active treatment was predictive of the positive effects on mood. This suggests the presence of positive *expectancy* biases in people self-administering low doses of psychedelics, an idea that has been further confirmed by a recent online survey (Kaertner et al. 2021). This is a recurrent problem in psychopharmacological research, especially with psychedelics, where patients are often able to break the placebo blinding due to the strong subjective properties of the drugs (Gukasyan and Nayak 2021). Taken together, these observations highlight the need to better establish the long-term effects of intermittent low dosing of psychedelics in both healthy and diseased populations, in order to assess whether the mystical experience is indeed necessary for the antidepressant effects of these drugs. Importantly, such studies should account for potential biases that may arise due to breaking of the study blinding shown in previous studies.

There are various ways this can be done. A simple solution would be to include only drug-naïve patients in trials investigating low doses of psychedelics, as these patients are likely to be less sensitive to detecting the subjective properties of these drugs (Kuypers 2020). Yet, there seems to be no difference between experienced and naive users in the positive expectations about the effects of intermittent low dosing of psychedelics (Polito and Stevenson 2019). Therefore, results could still potentially be biased in case of breaking the blinding by inexperienced users, and other solutions should be preferred. For example, examination of the nature of the subjective effects of low doses of psychedelics using drug discrimination studies and development of an active placebo that mimics these effects. Unfortunately, to our knowledge, there are no psychedelic drug discrimination studies with human subjects, whereas numerous drug discrimination studies are available for nonhuman subjects as described in “Drug discrimination of psychedelics” section. Given the high correlation between the discriminative stimulus and the subjective effect, the drug discrimination assay is an excellent tool for evaluating the true nature of the subjective effect in humans.

A second potential approach to reduce biases in the study results of RCTs with psychedelics is to evaluate psychedelic effects with more objective measures, such as cognitive outcomes rather than self-reported mood improvements. The idea is that cognition is less susceptible to expectancy biases that may originate due to breaking the blind (Schwarz and Büchel 2015). This was also demonstrated by the study of Szigeti et al. (2021) where the slight improvements in cognitive functioning induced by low doses of psychedelics were not influenced by the positive expectations of the participants. We have previously provided a rationale for the role of cognitive flexibility and PS improvements in the antidepressant effects of psychedelics (Magaraggia et al. 2021). Investigating improvements in PS and cognitive flexibility could therefore represent excellent behavioral outcomes because of their high translational value (Brown and Tait 2016; Robbins 2017; van Goethem et al. 2018). In rodents, PS and cognitive flexibility are often measured with the object pattern separation (OPS) task and attentional set-shifting task (ASST), respectively. Highly similar tasks are used in humans; thus, these behavioral measures are excellent tools for our translational model (Brown and Tait 2016; van Goethem et al. 2018). The use of such tasks would therefore provide an alternative to currently used measures of antidepressant-like effects in rodents (e.g., FST) that lack construct validity and perhaps translatability. However, individual differences and pronounced learning effects may present as confounding factors when using these tasks. There are two possible ways to account for these confounding factors. Firstly, the task must have multiple alternate test forms. Secondly, a within-study design can be used in order to eliminate these confounding factors. For example, Doss and colleagues (2021) studied the effects of psilocybin on cognitive flexibility in depressed patients. They had an initial 8-week baseline period before starting psilocybin treatment. If any changes were observed in cognitive flexibility, then these were regarded as non-treatment related changes, like learning or expectation biases (Doss et al. 2021). Moreover, we also argue that positive effects on these functions are visible in a time-dependent manner. Often people perform poorly in cognitive tasks during the acute effects of psychedelics, especially at high doses. Yet, the long-term effects of psychedelics on cognitive domains may be beneficial because the underlying stimulatory effects on neurogenesis and neuroplasticity may require time to become functional. For example, improvements in ASS may be most pronounced after the effects on structural neuroplasticity in the mPFC have reached their maximum, which we predict to be during the first week after the start of treatment based on previous studies (Ly et al. 2021). A similar rule might apply for neurogenesis-dependent improvements in PS. These may in fact require several months after initial stimulation to allow newborn neurons to proliferate, differentiate, migrate, and integrate into the hippocampal network before becoming fully functional (Denoth-Lippuner and Jessberger 2021). The latter effect might explain why in the RCT with psilocybin from Carhart-Harris and colleagues (2016), the antidepressant effects of psilocybin persisted at the 3-month follow-up. Yet, there is insufficient data to strongly support these claims. Therefore, there is a need for additional research that investigates the time-dose-dependent antidepressant effects of psychedelics with regard to neurogenesis and neuroplasticity mechanisms in rodents. Finally, another interesting and novel approach to test the role of the acute mystical and psychedelic properties is to investigate the antidepressant effects of 5-HT_2A_ agonists that can induce neuroplasticity without the subjective effects. These non-psychedelic ligands include LSD derivatives, such as the anti-Parkinson drug lisuride. Interestingly, there is preliminary evidence for the neuroplasticity-inducing effects of this drug in rodents (Olson 2018), and antidepressant effects in humans (Hougaku et al. 1994). Moreover, novel ligands have recently been developed based on the chemical structures of classical psychedelics that have been shown to stimulate cortical neuroplasticity, while lacking activity in the HTR assay (Cameron et al. 2020). We suggest further investigations with these drugs to better establish the role of the acute psychedelic and mystical experience in the antidepressant effects induced by psychedelics.

A final but important consideration pertains to the role of psychotherapy. As mentioned above, whereas psilocybin has so far mostly been administered in the form of PAP, ayahuasca was always given as a stand-alone treatment. On the contrary, the antidepressant effects of psychedelic “microdosing” have rarely been viewed in the context of psychotherapy. If the aim is to establish a dose–response relationship where the efficacy of high psychedelic doses is compared to the intermittent administration of low non-psychedelic ones, these should be administered following similar (psycho)therapeutic protocols. We believe psychotherapeutic support to be important for therapeutic success, as the underlying effects of neuroplasticity and cognition should be viewed in the context of psychotherapy (see “The role of the acute hallucinogenic and mystical experience in the antidepressant effects of psychedelics” section). Stand-alone pharmacological treatments for depression show small to moderate effect sizes, that substantially increase when combined with psychotherapy (Luoma et al. 2020). We assume that the window of cognitive flexibility induced by these agents may be beneficial especially for the creation and integration of novel cognitive-behavioral strategies needed by depressed patients to overcome the *negativity bias*. These strategies can be informed and taught by trained specialists through psychotherapy, and we agree with Nayak and Johnson (2021) that third-wave cognitive and behavioral therapies, such as Acceptance and Commitment Therapy, may be good candidates. Yet, more research is needed to establish the best psychotherapeutic protocol for these pharmacotherapies.

## Concluding remarks

Psychedelic medicine research is still in its infancy and there are still many unanswered questions regarding how these drugs work at the molecular, cellular, structural, and behavioral level. In this review, we briefly discussed the antidepressant effects of psychedelics and tried to test the relevance of the acute and psychedelic experience on these effects using a pharmacological translational framework. However, current evidence for the antidepressant effects of psychedelics has received various criticisms, and therefore more studies are needed to confirm these preliminary findings. Contrary to the canonical drug discovery and development pipeline, psychedelic research offers the possibility for a reverse translation of clinical human findings into preclinical models to investigate their underlying biological effects. In turn, the results of these studies can be utilized to further optimize current psychedelic treatment, for example by identifying molecular pathways responsible for the antidepressant and adverse effects of these drugs and develop novel molecules with an improved therapeutic window (i.e., non-psychedelic ligands). We believe that such an approach has the potential of bringing advancements in a field that has struggled to provide new solutions to long-existing problems.

## Abbreviations

5D-ASC: 5-Dimensional Altered States of Consciousness
5-HT2A: Serotonin 2A
5-HT: 5-Hydroxytryptophan
8-OH-DPAT: 8-Hydroxy-2-(di-n-propylamino) tetralin
ASS: Attentional set-shifting
ASST: Attentional set-shifting task
BP: Blood pressure
CD: Cluster of differentiation
CRP: C-reactive protein
DMT: *N*,*N*-Dimethyltryptamine
DOI: 2,5-Dimethoxy-4-iodoamphetamine
DOM: 2,5-Dimethoxy-4-methylamphetamine
DOI: 2,5-Dimethoxy-4-iodoamphetamine
ED50: Effective dose 50
FST: Forced swim test
HPA: Hypothalamic–pituitary–adrenal
HR: Heart rate
HRS: Hallucinogenic rating scale
HTR: Head twitch response
IL: Interleukin
LSD: Lysergic acid diethylamide
MAOIs: Monoamine oxidase inhibitors
MDD: Major depressive disorder
MDMA: 3,4-Methylenedioxymethamphetamine
MEQ30: 30-Item mystical experiences questionnaire
mGluR: Metabotropic glutamate receptor 2
mPFC: Medial prefrontal cortex
NK: Natural killer
NMDA: *N*-Methyl-d-aspartate
OPS: Object pattern separation
PAPs: Psychedelic-assisted psychotherapies
PD: Pharmacodynamics
PET: Positron emission tomography
PFC: Prefrontal cortex
PK: Pharmacokinetic
PS: Pattern separation
RCT: Randomized controlled trials
RDoC: Research Domain Criteria
SSRIs: Selective serotonin reuptake inhibitors
TCA: Tricyclic antidepressants
TRD: Treatment resistant depression
VAS: Visual analogue scales
WCST: Wisconsin Card Sorting Task
